# Molecular Changes in Cells of Patients with Type 2 Diabetes Mellitus Depending on Changes in Glycemia Level in the Context of Lifestyle—An Overview of the Latest Scientific Discoveries

**DOI:** 10.3390/cimb45030126

**Published:** 2023-02-28

**Authors:** Magdalena Szczechla, Anita Balewska, Dariusz Naskręt, Dorota Zozulińska-Ziółkiewicz, Aleksandra Uruska

**Affiliations:** Department of Internal Medicine and Diabetology, Poznan University of Medical Sciences, ul. Mickiewicza 52, 60-834 Poznań, Poland

**Keywords:** type 2 diabetes, glucose fluctuations, hypoglycemia, hyperglycemia, modifiable factors, transcriptional factors

## Abstract

Diabetes mellitus is a significant health problem for medicine and economics. In 80–90% of cases, it is type 2 diabetes (T2DM). An essential aspect for people with T2DM is to control blood glucose levels and avoid significant deviations. Modifiable and non-modifiable factors influence the incidence of hyperglycemia and, sometimes, hypoglycemia. The lifestyle modifiable factors are body mass, smoking, physical activity, and diet. These affect the level of glycemia and impact molecular changes. Molecular changes affect the cell’s primary function, and understanding them will improve our understanding of T2DM. These changes may become a therapeutic target for future therapy of type 2 diabetes, contributing to increasing the effectiveness of treatment. In addition, the influence of external factors (e.g., activity, diet) on each domain of molecular characterization has gained importance towards a better understanding of their role in prevention. In the current review, we aimed to collect scientific reports on the latest research about modifiable factors connected with the style of life which affect the glycemic level in the context of molecular discoveries.

## 1. Introduction

Diabetes mellitus (DM) is a fundamental health problem for medicine and socioeconomics. According to The World Health Organization data, the number of patients who suffer from DM is consistently growing—from 108 million in 1980 to 422 million in 2014 [1]. Moreover, the global diabetes prevalence in 2019 is estimated at 463 million people and is predicted to grow to 578 million in 2030 and 700 million in 2045 [2]. The constant increase in the number of patients makes diabetes the most common non-infectious disease in the world [2] and the second, after ischemic heart disease, in generating the most significant economic costs in society [3].

Type 2 diabetes (T2DM) accounts for 80–90% of diabetes cases [3]. This type of DM is a chronic disease manifested by the elevated level of glucose in the blood resulting primarily from insulin resistance which over time is joined by a defect in insulin secretion [4].

Environmental factors such as the patient’s lifestyle, smoking, lack of physical activity, and improper diet lead to obesity [4,5]. This influences the course of the disease, the morbidity, and the occurrence of hyperglycemia and hypoglycemia in patients with T2DM [6]. Fluctuations in the level of glycemia are reflected in molecular changes in cells. Understanding the molecular changes in cells contributes to a better understanding of T2DM. The aim of this study is to review the findings of molecular changes described in the literature in 2017–2022, occurring under the influence of changes in glycemia in patients with type 2 diabetes, with particular emphasis on how these changes result from lifestyle and what preventive and therapeutic challenges they create. We realize that this is an extensive area of research that cannot be covered well enough in one publication, which is why we decided to focus on examples of how modifiable factors affect glucose levels and what molecular changes they bring.

## 2. Lifestyle Factors Affecting Blood Glucose Level in the Context of Molecular Discoveries

The factors affecting glucose fluctuations can be divided into modifiable and unmodifiable. Many modifiable factors influence increases and decreases in blood glucose levels, which is positive information for clinicians dealing with T2DM [7]. In this review, the most important factors to focus on are diet, body mass, smoking, and physical activity. The available literature shows that they play a significant role in cell changes in people with impaired glycemia. A summary of this section presents in Table 1.

In addition, in the latest literature, there are modifiable factors such as the model of care, insurance structure, or drug therapy. These factors affect the course of the disease but are not measurable in the molecular context. Therefore, despite their importance, they will not be included in this work [7].

The non-modifiable factors in the current literature include age, longer duration of diabetes, sex, family history, or gestational diabetes [8,9,10]. In our work, however, we deal strictly with modified causes.

**Table 1:** The molecular consequences of factors affecting glucose level.

Factor	Molecular Cause	Metabolic Response
**Body mass**	“macrophages impair the ability to secrete insulin by β cells” [11]	“insulin resistance leads to hyperglycemia” [12]
	“fluctuations of blood glucose levels are promoting inflammation caused by the accumulation of circulating SFA” [13]	“lipotoxicity” [13]
	“translocase CD36 as a FA transporter” [14]	“a key molecule in the reduced insulin within higher body weight” [14]
	“the occurrence of low blood sugar after RYGB” [15]	“moderate hypoglycemia impairs neurological function; severe hypoglycemia leads to the death of neurons” [16] “hypoglycemia may cause a proarrhythmic effect” [17]
	“calcium-sensitive kinase, calcium/calmodulin dependent-protein kinase II (CaMKK), plays a critical role in glucose homeostasis” [18,19,20]	“CaMKK regulates insulin signaling and glucose homeostasis throughout the body and in lipolysis and adipocyte inflammation.” [21]
**Smoking**	“nicotine determinate aging of β cells of the pancreas” [22]	“impaired glucose metabolism, higher glucose variability and worse course of previously diagnosed T2DM” [22]
	“exposure to nicotine changed the expression of aging proteins such as p16, p19, p21 when exposed to higher values” [22]	
	“nicotine exposition might lead to hdl accumulation in pancreatic islets cells”	
	“gene TCF7L2 was densely expressed in the mHb region of the rodent brain and regulated the function of nicotinic acetylcholine receptors” [23]	“nicotine-induced increase in blood glucose may affect the response of mHb neurons and stop local NACHR function causing habitual tobacco smoking” [23]
	“increased circulating glucose levels can modulate mHb function” [23]	
**Physical activity**	“the elevation of the plasma levels of extracellular HSP70 is connected to obesity and diabetes” [24]	“reduced extracellular HSP70 concentration can inhibit inflammation, mitochondrial fatty acid oxidation, and increase activation of SREBP-1c, which is a gene transcription factor in ER stress” [25]
	“increased HSP70 expression in brain cells can increase insulin sensitivity, normalize blood glucose level” [24]	
	“long-term exercises increased the production of intracellular HSP70 in the muscle, the liver, kidneys, and heart” [26]	
	“HSP72 has exercise-induced expression” [27]	“reduce the pro-inflammatory cytokines, increase insulin sensitivity” [27]
	“intracellular HSP-72 is anti-inflammatory by blocking the activity of the jnk and nk-kb pathways” [27]	
	“physical activity in connection to an increase in tissue temperature that occurs during exercise can increase HSP72 concentration” [27]	
	“in human skeletal muscles is noted two proteins of the orphan nuclear receptor family, Nur77 and NOR1” [28]	“induce insulin response genes and glucose and fat metabolism” [29]
	“aerobic exercise strongly increased Nur77 and NOR1 in a healthy population” [30]	
	“diabetes can lead to increased levels of tau protein and Aβ” [31]	“HIIT significantly reduced blood glucose and tau and Aβ protein levels.” [32]
**Diet**	“there is no difference in HbA1c decrease in the Mediterranean diet compared a low-fat diet. there was a significant decrease in fasting glucose concentration in the Mediterranean group compared to the low-fat diet” [33]	“it can be concluded that the fluctuations in both hyperglycemia that make up the mean HbA1c score are reduced in the Mediterranean diet” [33]
	“human islet amyloid polypeptide—hIAPP is probably responsible for the loss of β cell function, and death” [34]	“the polyphenols found in virgin olive oil have antioxidant properties and the ability to inhibit the growth of human islet amyloid polypeptide—hIAPP” [35]
	“lipotoxicity can disrupt Cx36 gap junctions couplings within the islets in diet-induced obesity” [36]	“decrease in insulin secretion and variability in glycemic homeostasis [36]
	“the mechanism of action of HFD contributing to the disorders of homeostasis is multidirectional.” [37]	“aggregation of Aβ and hyperphosphorylated Tau protein” [37] “chronic overconsumption of HFDs leads to impaired glucose homeostasis, insulin resistance and T2DM” [38]

### 2.1. Body Mass

The low-grade inflammation in metabolic diseases, including obesity and diabetes, is widely described in the literature [39]. There is ample evidence that inflammatory processes, in addition to adipose tissue and the liver [40], are also activated in pancreatic islet cells in obese patients [11]. Increased local proliferation of macrophages in islets provides signals of promoting β-cell hyperplasia [41]. Macrophages impair the ability to secrete insulin by β cells [11], thus enhancing insulin resistance, leading to hyperglycemia [12]. Moreover, fluctuations between normal and high blood glucose levels promote inflammation caused by the accumulation of circulating saturated fatty acids (SFA), leading to lipotoxicity [13]. Lipotoxicity may impair the function of β-cells and induce apoptosis [42]. Thus, this is a vicious circle mechanism obesity, which causes inflammation through blood glucose fluctuations, and hyperglycemia causes even more inflammatory damage, which causes greater increases in blood glucose. So, reducing body weight and therapeutic tools targeting islet inflammation may help restore normal β-cell function and reduce hyperglycemia in patients [43]. It is possible that working on reducing inflammation will achieve another effect in the form of reducing glycemic fluctuations and, as an added benefit, helping patients to reduce body weight more effectively, simultaneously destroying the vicious circle. The abovementioned β-cell lipotoxicity, known as the accumulation of lipids in β-cells, is observed with increased Body Mass Index (BMI) [44]. It considers that both endogenous fatty acids (FA) synthesis and FA uptake are important for the increased accumulation of lipids in islets [45]. Nagao et al. compared expression levels of the FA transporters in islets and paid attention to the role of facilitated FA uptake for defective insulin secretion. They tested the hypothesis that translocase CD36 as a FA transporter can be a key molecule in the reduced insulin secretion capacity in a population with higher body weight [14]. The main function of CD36 is to facilitate an influx of long-chain SFA (LCSFA) across the plasma membrane, whose harmful effects on islets are widely described in the literature [46]. In the study, overweight patients with T2DM expression of CD36 in islets was 70% higher than obese patients but without T2DM, showing a clear correlation that overexpression of CD36 in human islets led to decreased insulin secretion. Overexpression of CD36 stops exocytosis by reducing exocytotic protein levels and reducing granule docking. However, impaired exocytosis does not fully explain the phenomenon of lipotoxicity. This finding demonstrates the molecular interrelations’ complexity, resulting in impaired insulin secretion in obese patients. The conception of CD36 antibody treatment of the human β-cell line EndoC-βH1 improves insulin secretion. Those results promise to prevent the hyperglycemia caused by insufficient insulin secretion developed in type 2 diabetes in obese people or change the natural course of an already developed disease by reducing glucose levels caused by insufficient insulin production over time. Such new drugs need further and long-lasting tests [14]. It seems ironic but similar results in limiting the inflammatory processes can be achieved in the simple idea of body weight reduction.

One of the newest obesity treatment interventions is bariatric surgery [47]. Zuo et al., in their studies, checked the long-term effectiveness of bariatric treatment and its influence on β-cells after three years of follow-up [48]. Among the patients enrolled in the study, body weight, HbA1c, glucose, and insulin levels decreased within six months and one year after surgery, but after three years, weight gain was observed on average by 31%. The improved islets function of the pancreas with weight loss and the worsened function during weight gain show that obesity impacts the ability to secrete insulin. Impaired secretion leads to insulin deficiency and, in consequence, increased glucose levels. The improvement was caused by a reduction in body mass and blood glucose levels, leading to a lower burden on β-cells and, at the same time, supporting the function of pancreatic islets bringing a positive impact on improving blood glucose [48]. Therefore, T2DM and glucose level incidence are related to body weight [49]. Although the mean glucose in the continuous glucose monitoring system was negatively related to BMI [50], body weight fluctuation was associated with micro and macrovascular complications in T2DM patients, regardless of the patient’s baseline body weight and BMI [50].

On the other hand, bariatric surgery such as Roux-en-Y gastric bypass (RYGB) surgery, sleeve gastrectomy, and fundoplication contribute to the occurrence of hypoglycemia [15]. The occurrence of low blood sugar after RYGB surgery is explained by a wider glycemic excursion after food consumption, with an earlier and higher glucose peak and a lower glucose nadir as a result of bypassing the pylorus and the proximal intestine [15,51,52]. In addition, early postprandial insulin and GLP-1 secretion increase due to the rapid delivery of nutrients to the proximal anterior segment. In parallel with the altered glycemic pattern, early postprandial insulin and GLP-1 secretion are excessive after RYGB due to the rapid delivery of nutrients to the proximal anterior segment [51,52,53,54]. Moderate hypoglycemia impairs neurological function, but severe hypoglycemia leads to the death of selectively vulnerable neurons [55]. Hypoglycemia induces neuronal depolarization. Depolarization leads to a release of glutamate, aspartate, and zinc. Impaired astrocyte uptake may contribute to increased glutamate extracellular levels. Glutamate receptor activation and zinc influx induce the production of reactive oxygen species (ROS) from mitochondria or NADPH oxidase, subsequent DNA damage and activation of poly (ADP-ribose) polymerase-1 (PARP-1), mitochondrial permeability transition (MPT), which leads to cell death [16,17]. Hypoglycemia is dangerous to nerve cells and contributes to cardiac dysfunction [56]. Hypoglycemia may cause a proarrhythmic effect [17]. Lowered glucose levels block the repolarizing K(+) channel HERG, which leads to an action potential and QT prolongation and is uniformly associated with risk for torsade de pointes ventricular tachycardia [57]. Hypoglycemia increases the sympathetic response and the risk of arrhythmias from Ca(2+) overload, which occurs with sympathomimetic medications and excessive beta-adrenergic stimulation [58,59,60]. It recommends minimizing fluctuations in body weight in patients with T2DM during weight loss to prevent complications resulting from the dominant disease and too-low or -high glucose levels. Fluctuations in body weight also impacted glucose variability [50]. Thus, the reduction of excess body weight should be gradual, stable, and effective to achieve a better and safer outcome.

Adipose tissue is now recognized as a vital endocrine organ that contributes to the energy homeostasis of the whole body. The role of calcium homeostasis in metabolic disorders has become a subject of interest in recent years. During fasting and obesity, a calcium-sensitive kinase, calcium/calmodulin dependent-protein kinase II (CaMKK), becomes activated in the liver and plays a critical role in glucose homeostasis [18,19,20].

Deletion or inhibition of hepatic CaMKK in obese mice protects against glucose intolerance and hyperinsulinemia [18]. This mechanism is related to the suppression of gluconeogenesis and the improvement of insulin signaling in the liver.

Similarly, CaMKK deficiency enhances skeletal muscle insulin receptor (INSR) signaling and glucose transport in diabetic mice [61]. Wen Dai et al. additionally presented the role of CaMKK in another insulin-sensitive tissue, adipose tissue. Previous studies on isolated human adipocytes have shown increased intracellular calcium concentrations and concomitant decreases in glucose uptake in obese subjects [62]. In drosophila, store-operated calcium entry regulator, stromal interaction molecule 1 (STIM1), and the endoplasmic reticulum–localized calcium channel, inositol triphosphate receptor (ITPR) have been implicated in regulating adiposity and lipid metabolism [63,64]. Wen Dai et al. showed that CaMKK is activated in obese adipose tissue [21].

Using cultured CaMKK-deficient adipocytes and an inducible mouse model of CaMKK deficiency in adipose tissue, he demonstrated that the CaMKK adipocyte regulates insulin signaling and glucose homeostasis throughout the body. In addition, CaMKK is involved in lipolysis and adipocyte inflammation, which may further contribute to the pathogenesis of insulin resistance. These data identify the CaMKK adipocyte as a critical component of metabolic regulation in obesity [21]. Thus, adipocyte-specific inhibition of CaMKK may be beneficial in metabolic dysfunctions such as hyperglycemia in type 2 diabetes.

### 2.2. Smoking

Cigarette smoking is another modifiable factor influencing glucose levels. On the one hand, smoking is a risk factor for the onset of T2DM. In the meta-analysis, Willi et al. show clear evidence that active smoking is related to up to 30–40% increased risk of T2DM [65] in active smokers compared to patients who have never smoked [66,67]. Moreover, Kim et al., in a presented group of 78,212 Koreans, showed that smoking, measured by a self-reported questionnaire and urine cotinine (nicotine metabolite), was related to the much more frequent diagnosis of new-onset diabetes mellitus (NODM) in this study named as having no DM but developing the disease at a follow-up. Likewise, they proved that actively smoking patients who smoked 20 and more cigarettes per day have a 64% higher risk of NODM, and patients who smoked for more than ten years have a 34% higher risk of NODM. The duration of exposure and the severity of nicotinism influenced the processes of pancreatic β-cell dysfunction [68]. Cigarette nicotine changes glucose metabolism and impairsinsulin influence on the liver, adipose tissue, and muscles. As a result, it may lead to hyperglycemia [69]. Moreover, heavy metals as components of cigarettes also affect the incidence of diabetes, especially exposure to arsenic, although the results of studies in the literature are inconsistent [70]. Clinical studies suggested that smoking impacts body composition, insulin sensitivity, and pancreatic β-cell function [70]. Until now, the mechanisms of pancreatic islet destruction in the context of nicotine exposure have remained elusive. In vitro studies on human cells and rodents provided an insight into the molecular mechanisms that, in the case of β cells, are extremely important in the context of the therapeutic potential of DM. Sun et al., in their newest study, showed for the first time that nicotine determines the aging of β cells of the pancreas both in vivo and in vitro, which affected impaired glucose metabolism, higher glucose variability, and worse course of previously diagnosed T2DM. Exposure to nicotine was dose-dependent and caused the apparent aging phenotype of β-TC-6 cells at lower concentrations and changed the expression of aging proteins such as p16, p19, and p21 when exposed to higher values. Moreover, Sun et al. revealed that intracellular Ca^2+^ and reactive oxygen species (ROS) levels were significantly elevated in β-TC-6 cells; therefore, nicotine exposition might lead to ROS accumulation in pancreatic islet cells. The expression of senescent markers and accumulation of ROS in β cells revealed that nicotine intake enhances the senescence of β cells. These changes may contribute to the progressive loss of islets and progressively more frequent fluctuations in blood glucose levels and gradually approaching diagnosis of T2DM [22]. β cell regeneration and mass are important in long-term glucose control. Thus, premature senescence of pancreatic β cells may contribute to smoking-induced T2DM [71]. Interestingly, Sun et al. noticed that nicotine-induced aging of β cells is devastating to glucose homeostasis in high-fat diet (HFD)-fed mice but not in mice with a normal diet. It hypothetically shows a connection between two modifiable factors that can be used as a lifestyle intervention that will work with redoubled force [22]. Medical intervention that protects β cells from aging may be a strategy for preventing smoking-associated diabetes development, which is compatible with reducing glucose variability and maintaining metabolic homeostasis [22]. Smoking cessation reduces the risk of developing diabetes [65], although ex-smokers have a higher risk of developing diabetes than those who have never smoked. Quitting smoking is incredibly profitable for patients because the risk of developing diabetes decreases with increasing smoking abstinence [72], although the initial effect of quitting smoking may be weight gain, most often in the central part of the abdomen, which may lead to impaired glucose tolerance, insulin resistance, and, consequently, diabetes. Quitting smoking is associated with a lower risk of diabetes in the long term [73]. Unfortunately, a study by Keith et al., who studied the rate of nicotine metabolism in people with diabetes compared to the healthy population, found that T2DM patients had an average 36.5% higher nicotine metabolism rate (NMR). People who metabolize nicotine faster are exposed to the tendency to smoke more for a longer period, which often leads to the inability to give up smoking and increases the health effects of smoking [74]. It should be mentioned that smoking is associated with insulin resistance and hyperglycemia in a dose-dependent manner. Even in healthy men, chronic smoking was connected to high insulin concentration and insulin resistance [75]. It is unclear whether T2DM causes higher NMR or whether people with higher NMR are at risk of developing diabetes [74]. Unfortunately, one thing is for sure: the effect of this relationship is the destruction of glycemic homeostasis and progressive hyperglycemia. Smoking cessation, not only in diabetes patients but in every addicted person, is the best choice and form of therapy to counteract the effects of glucose metabolism disorders. Duncan et al. showed that the diabetes-associated gene TCF7L2 is densely expressed in the rodent brain’s medial habenula (mHb) region. It regulated the function of nicotinic acetylcholine receptors (nAChR) [23]. Gene TCF7L2 inhibition was responsible for increasing nicotine consumption in rodents, while blood glucose levels were increased by nicotine through TCF7L2-dependent stimulation of the mHb mechanism. Duncan developed that the pancreas has a polysynaptic connection to the mHb, so nicotine intake correlated with increased glucose and insulin level, similar to unregulated homeostasis due to T2DM development. Increased circulating glucose levels can modulate mHb function. Nicotine-induced increases in blood glucose may affect the response of mHb neurons and stop local nAChR function while causing habitual tobacco smoking. Frequent fluctuations in glycemia and hyperglycemia in T2DM patients cause mHb function modulation, inhibiting nAChR and enhancing addiction. That mechanism could explain in another way why T2DM patients have a more serious quitting problem and are frequent smokers. Moreover, mHb probably regulates hyperglycemic response to stress as a component of fight-or-flight behavior. During nicotine fasting, mechanisms analogous to a state of hunger are presented. By repeatedly hijacking this mHb-regulated stress response, frequent nicotine intake reveals abnormalities in blood glucose levels in a TCF7L2-dependent manner. As mentioned above, loss of TCF7L2 function causes increased nicotine consumption but decreases hyperglycemic response to nicotine and protects against disturbing glucose level homeostasis. If this fact extends to human smokers, it can show the mechanism that deficiency in TCF7L2-dependent signaling leads to an increased risk of tobacco addiction but simultaneously protects against smoking-related diabetes [23]. This finding suggests that DM may initiate in the brain. The discovery’s conclusions open new perspectives for research to protect patients against nicotine-induced changes in habenula-regulated interactions with the anatomic nervous system. It indirectly shows that focusing on molecular mechanisms taking place only in the pancreas is insufficient, as DM is a systemic disease that is also reflected in the central nervous system. Thus, glucose variability affects the communication pathways between the pancreas and mHb, exacerbating the problem of smoking and decompensation of the underlying disease.

### 2.3. Physical Activity

The following recognized modifiable factor for glucose control is physical activity. It is recommended for people of all ages, regardless of their health condition, as it is perceived as a factor leading to organism homeostasis. Undoubtedly, physical activity reduces the probability of T2DM by increasing insulin sensitivity and glucose tolerance [76], leading to the prevention of hyperglycemia. Skeletal muscles constitute a specific glucose store and a metabolic factory [77]. Maintaining the correct mass of skeletal muscles, understood as the balance between protein synthesis and protein breakdown [78], is a safety buffer for proper glycemic management. T2DM, in an unclear mechanism, causes atrophy of skeletal muscles and loss of nuclei [79,80]. The reduction in insulin reactivity of the mammalian target of rapamycin complex 1 (mTORC1), which is a regulator of muscle protein synthesis, was considered the cause [81]. Ato et al., in their study, assessed the connection between T2DM and muscle growth under the influence of exercise corresponding to resistance training (RT) in rodents. Although the number of muscle nuclei and mTORC1 activity deteriorated in the T2DM rats compared to the control group, the muscle mass affected by RT was similar in both groups. Therefore, physical exercise and associated muscle mass changes are an intervention that improves the patient’s health, regardless of the advancement of T2DM [82]. In the study by Lindström et al., in people with impaired glucose tolerance, introduction of lifestyle changes consisting of weight loss, consumption of saturated fat, increased dietary fiber consumption, and increased physical activity led to a reduction in T2DM incidence and permanent changes in the way of life of patients [83]. There is a positive correlation between BMI and a significant negative correlation between physical activity and T2DM development [84].

Nowadays, the molecular background of this relationship is investigated. Heat shock proteins (HSPs) play an essential role in the pathogenesis of insulin resistance, which leads to hyperglycemia, for example, Heat shock protein 70 (HSP70) [24,85]. HSP70 is a cytoprotective chaperone that folds and degrades proteins. There is an association between this molecule’s induction, transcription, and translation and a decrease in insulin resistance-related metabolic diseases through inflammation, mitochondrial function, and endoplasmic reticulum (ER) stress [25]. The elevation of the plasma levels of extracellular HSP70 is connected to obesity and diabetes, which are pro-inflammatory states mentioned in the current review. Reduced HSP70 concentration can inhibit inflammation and mitochondria fatty acid oxidation, and increase activation of SREBP-1c, a gene transcription factor in ER stress. Increased HSP70 expression in brain cells can increase insulin sensitivity and normalize blood glucose levels.

The investigation in well-characterized animal models to represent the human condition with T2DM showed that long-term exercises [86,87] increased the production of intracellular HSP70 in the muscle, the liver, kidneys, and heart [26], and can blunt the partial increase of insulin resistance in the whole body. Thus, long-term exercises through better insulin sensitivity decrease glucose variability and make the constant metabolic conditions a beneficial effect on whole-body homeostasis. Another HSP, heat shock protein 72 (HSP72), also has exercise-induced expression. Intracellular HSP72 is anti-inflammatory by blocking the activity of the JNK and NK-κB pathways and reducing the pro-inflammatory cytokines, increasing insulin sensitivity. The concentration of HSP72 is decreased in skeletal muscle and liver in patients who suffer from obesity and insulin resistance compared to the healthy group. Moreover, the overexpression of HSP72 in skeletal muscle can prevent a high-fat diet-induced glucose intolerance and insulin resistance in rats [88]. Physical activity in connection with an increase in tissue temperature during exercise can increase HSP72 concentration and benefit glycemia [27]. Thus, through well-chosen exercises maintaining an appropriately high temperature of the patient’s body, ensuring an increase in HSP72, it is possible to generate appropriate metabolic pathways that will reduce the negative effects of both obesity and type 2 diabetes, reducing hyperglycemia or even leading to normoglycemia. In the research presented by Atkin et al., there was a group of heat shock and related proteins that, overall, tended to be consistently higher in T2D compared to controls in response to hypoglycemia [89]. In addition, a significant increase in interleukin-6 was observed both in the control group and in T2D patients 4 h after induced hypoglycemia [89].

In recent decades, microarray analysis has allowed the consideration of many potential therapeutic targets. In human skeletal muscles, researchers noted two proteins of the orphan nuclear receptor family, Nur77 and NOR1 [28], which induce insulin response genes and glucose and fat metabolism [29]. Pearen et al., in their work, suggested that aerobic exercise, which offers the benefit of increasing insulin sensitivity and improving glucose and lipid metabolism, might be related to NOR1 [90]. Aerobic exercise strongly increased Nur77 and NOR1 in a healthy population. Mey et al. proved that in both obesity and T2DM, the insulin response regulated by Nur77 and NOR1 was impaired, and interventions in the form of aerobic exercise partially restored this response [30]. If the presented hypothesis is correct, the activation of NOR1 is responsible for regulating exercise adaptations; then, it is possible to use activating NOR1 function in treating metabolic diseases, including diabetes. It raises the possibility of pharmacological enhancement of NOR1 while enhancing the positive effect of exercise on diabetes-related hyperglycemia.

Diabetic patients suffer from anxiety and depressive symptoms more often and are more likely to develop dementia than healthy people of the same age and gender [91,92,93]. The high comorbidity of obesity and psychiatric disorders such as anxiety can exacerbate metabolic and neurological symptoms dramatically. Orumiyehei et al. investigated the effects of high-intensity interval training (HIIT) on molecular brain changes and cognitive and anxiety behaviors in type 2 diabetic rats [32]. This study showed an increase in Tau and Aβ protein levels in the hippocampus of diabetic rats. Accumulations of hyperphosphorylated tau protein and amyloid beta (Aβ) lead to memory impairment, which is the hallmark of Alzheimer’s disease (AD), the leading cause of dementia [31]. As previous studies have shown, diabetes can lead to increased Tau protein levels and Aβ in humans and animals [94]. HIIT significantly reduced blood glucose and tau and Aβ protein levels. Induction of anxiolytic behavior has also been observed. The result of the study indicates the neuroprotective effects of exercise in diabetes-induced brain disorders.

At this point, attention should also be paid to another effect of sugar levels on the nervous system. Hypoglycemia also has an adverse effect on its operation. A study presented by Zhou et al. found disruption of mitochondrial homeostasis in the hippocampal tissue of diabetic mice experiencing repeated mild hypoglycemia. Hypoglycemia disrupted the fine mitochondrial structure, reduced the number of mitochondria, and upregulated the expression of mitochondrial dynamics and mitophagy markers, including dynamin-related protein 1 (Drp1), Bcl-2/adenovirus E1B 19-kDa-interacting protein-3 (BNIP3), and microtubule-associated protein 1 light-chain 3 (LC3) in the hippocampus of T1DM mice [95]. In subsequent studies, diabetic mice subjected to severe hypoglycemia showed histological damage, damage to the blood–brain barrier, cerebral edema, and loss of pericytes. There was also a decrease in the expression of BBB tight junction proteins occludin and claudin-5, expression of markers specific for pericytes PDGFR-β (platelet-derived growth factor receptor-β) and α-SMA, and an increase in the expression of the inflammatory factor Matrix Metalloproteinase-9 (MMP9) [96]. This resulted in cognitive impairment in the tested mice.

### 2.4. Diet

The last featured factor that influences glucose variability is diet. The goals of introducing a diet in patients with T2DM are based on well-introduced dietary habits, which can eliminate the primary symptoms of the disease, reduce the risk of glucose fluctuations, and thus eliminate micro and macrovascular complications [8]. The meta-analysis by Esposito et al. stated that all diets recognized by current science as healthy diets equally reduced the risk of T2DM by 20% [97]. Good dietary intervention can reduce long-term changes caused by hyperglycemia and complications caused by T2DM and stop the progression of T2DM or lead to the remission of the disease [98]. Generally known dietary recommendations are lower lipid intake because excessive lipids increase cholesterol, triglycerides, and free fatty acids, which may impair metabolic function viability [36,99]. HFD, after all, is a factor often used in molecular studies to obtain lipotoxicity, which was mentioned in the current review.

As mentioned earlier in this paper, diabetes is a factor in Alzheimer’s disease. A HFD contributes to the development of glucose homeostasis disorders [100]. HFD stimulates diabetes and insulin resistance in neuronal Thy1-C/EBPβ (Tg) transgenic mice, accompanied by a marked accumulation of mouse Aβ and hyperphosphorylated Tau aggregation in the brain, inducing cognitive deficits. This effect is weakened after deleting AEP from C/EBPβ Tg mice [101]. Additionally, exposure to HFD often results in insulin resistance and pancreatic β-cell dysfunction. The mechanism of action of HFD contributing to the disorders of insulin homeostasis is multidirectional. HFD causes attenuation of β-cell insulin secretion and peripheral insulin action through inflammation of the hypothalamus [37]. Pro-inflammatory cytokines produced by the β cells due to islet macrophage accumulation may further block β cell function [102]. In addition, HFDs can activate 12-lipoxygenase activity in β-cells through cytokines, FFA, and increased blood glucose levels, thereby increasing the production of 12-hydroxyeicosatetraenoic acid (12-HETE). 12-HETE promotes oxidative stress and impairs Nrf2 function, leading to β-cell apoptosis and glucose intolerance [103]. This contributes to the increased insulin release from the β-cells and thus to hyperinsulinemia. Hyperinsulinemia coupled with an inflammatory response promotes lipolysis in adipose tissue, allowing FFA and glycerol to flow into the liver and promote gluconeogenesis. At the same time, increased insulin levels increase muscle glycolysis and lactate production, which is released into the circulation and can be used as a substrate for gluconeogenesis in the liver [104]. Subsequently, increased gluconeogenesis increases hepatic glucose production and thus leads to systemic insulin resistance. In addition, recent studies have shown that the A2b adenosine receptor (A2bAR), a recognized mediator of inflammation, controls pancreatic dysfunction in HFD-induced obesity [38]. In summary, chronic overconsumption of HFDs leads to impaired glucose homeostasis, insulin resistance, and T2DM.

A high level of SFA is a pro-inflammatory factor and leads to impairment of β-cells function and, as a result, loss of glycemic control [33,105,106]. A diet with a lower carbohydrate content enables the reduction of HbA1c reduction in people with previously diagnosed diabetes [107]. However, compared to a lower-carbohydrate diet, the Mediterranean diet significantly reduces HbA1c [106]. Moreover, there is no difference in HbA1c decrease in the Mediterranean diet compared to a low-fat diet. Interestingly, there was a significant decrease in fasting glucose concentration in the Mediterranean group compared to the low-fat diet [33]. Thus, given that HbA1c is the mean glucose value and fasting glucose is much lower, it can be concluded that hyperglycemia and hypoglycemia that make up the mean HbA1c score are reduced in the Mediterranean diet. A hypothetically similar HbA1c value in low-fat diets may equate to more significant rises and falls in blood glucose levels during the day. This effect is not seen as an increase in HBA1c as it is still the average of 3-month blood glucose levels. Perhaps that is why the diet recommended in the literature is the Mediterranean diet, which seems to be the most accurate dietary recommendation. The assumptions of the Mediterranean diet are based on the content of plants and oils. The polyphenols found in virgin olive oil have antioxidant properties and the ability to inhibit the growth of human islet amyloid polypeptide-hIAPP [35], which is probably responsible for the loss of β-cell function and death [34]. There are no therapeutic strategies to prevent amyloid aggregation. Therefore, consuming olive oil in T2DM patients appeared to have a high benefit balance. The mechanism of action of polyphenols is related to alleviating inflammation and oxidative stress, partly by regulating the AMP-activated protein kinase (AMPK) and nuclear factor-κB (NF-kB) signaling pathways [108]. Altered activation of AMPK by phosphorylation is associated with inflammation in a mouse model of obesity or obese patients [109,110]. One of them, ferulic acid, reduces oxidative stress and inflammation throughout the body, as evidenced by reduced production of ROS, pro-inflammatory cytokines, and expression of adhesion molecules and circulating low-density lipoprotein (LDL) levels by upregulating AMPK phosphorylation [111]. Additionally, adiponectin expression and circulating high-density lipoprotein (HDL) levels are increased [111]. In the studies presented by Huang et al., resveratrol, by influencing AMPK signaling, reverses mitochondrial dysfunction, increases total antioxidant capacity, increases superoxide dismutase SOD and glutathione peroxidase (GPx) activity, and reduces the content of malondialdehyde (MDA) and carbonyl protein in obese mice fed with HFD [112]. Consistent with these observations, resveratrol inhibits NF-κB activation, resulting in reduced tumor necrosis factor-α (TNF-α), interleukin-1β (IL-1β), interleukin-6 (IL-6), and cyclooxygenase 2 (COX-2) mRNA expression and reduced secretion of IL-6 and prostaglandin E2(PGE2) in adipocytes [113]. In summary, phenolic compounds may modulate oxidative stress and obesity-related inflammation through various mechanisms: NADPH oxidase (NOX), AMPK, NF-κB, protein kinase C (PKC), and Nrf2 signaling most likely mediate. Therefore, targeting these pathways may be a practical therapeutic approach for treating metabolic disorders such as insulin resistance. In addition to the abovementioned properties, olive oil is of great interest due to its biological properties, having also anti-cancer, antibacterial, and protective properties for erythrocytes, being a source of vitamins (tocopherol), polyunsaturated fatty acids (FFA), and about thirty natural phenolic compounds, including oleuropein (Ole), which has proven antidiabetic activity, by inhibiting the cytotoxicity induced by hIAPP aggregates [114]. Cooking oils, including olive oil, also contain FFA, which, depending on their saturation, has a positive or negative effect on the functioning of β cells, thus increasing the risk of developing T2DM. In their work, Von Hanstein et al. investigated the toxicity effect on pancreatic β cells of certain mixtures of unsaturated and saturated fats [115]. The results were surprising because they determined that the more prominent component of unsaturated FFA in oil compositions did not protect against T2DM development. Only the chain length of the FFA accounted for the toxicity of the FFA to EndoC-βH1 cells. Medium-chain FFA shorter than C16 were not toxic to EndoC-βH1 cells [115]. Such chain lengths are found in palm kernel and coconut oil. The researchers, therefore, concluded that the unique positive effect of consuming olive oil was not associated with a large amount of unsaturated fatty acids, which was commonly repeated information [115,116], but with the presence of other plant components, such as the polyphenols described above [115]. Dietary recommendations in the form of consumption of olive oil are still valid and justified by the protection of pancreatic cells against death, and thus worse glycemic homeostasis and frequent fluctuations leading to the diagnosis of T2DM.

In addition to the Mediterranean diet, there are various dietary ideas to improve health. Bearing in mind that T2DM is also influenced by changes in the composition of macronutrients, regulating metabolic functions, glucose homeostasis, and influencing the biology of pancreatic cells [117,118], Her and colleagues tested the hypothesis of the effect of carbohydrates on the modulation of the energetic and metabolic adaptation of pancreatic islets to high-fat diets on mouse models. The significant restriction of carbohydrates in the diet typical of ketogenic diets reduced the amount of adipose tissue, increased energy expenditure, reduced glycemia, and insulin resistance compared with high-fat and high-carbohydrate diets. Significant reduction in carbohydrates in the diet impacted molecular changes in pancreatic islets, which is highlighted by a limited capacity for β-cell mass expansion in response to HFD, characterized by a change in the secretory response. The ketogenic diet is certainly a diet that reduces the development of obesity. However, more research is needed on the effects of this diet on β cell biology to introduce specific dietary restrictions to the care of patients with T2DM [119]. In addition, caloric restriction can reduce the incidence of metabolic diseases, although the mechanisms behind this benefit are not fully understood. Do Amaral and colleagues investigated whether caloric restriction could protect against a decrease in insulin secretion and variability in glycemic homeostasis, for which intercellular communication inside the islets of Langerhans via the Connexin36 (Cx36) gap junctions was responsible. Rodents showing signs of pre-diabetes after a high-fat diet, after a monthly 40% caloric restriction, showed a decrease in body weight and regained insulin sensitivity. Lipotoxicity can disrupt Cx36 gap junction couplings within the islets in diet-induced obesity. Thus, the decrease in Cx36 gap couplings that typically occur in metabolic diseases could be regenerated by caloric restriction, improving Ca^2+^ dynamics, and regulating insulin secretion. Therefore, limiting the caloric supply may be a factor that will prevent patients from developing metabolic diseases, including obesity and T2DM, and in people with diabetes, it can decrease glucose variability [36].

## 3. Summary

**Figure 1:**
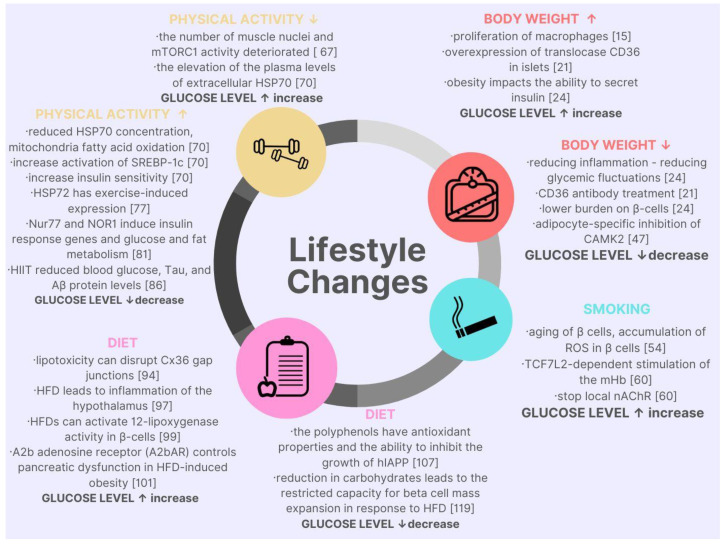
Graphic summary of lifestyle changes in the context of molecular cell changes and their impact on glycemic levels.

The current review of the latest research proved that the modifiable factors affecting glucose variability, hypo-, and hyperglycemia are significant in the development and progression of T2DM. The interventions in body mass, smoking, diet, and physical activity in diabetic patients impact the course and prognosis of the disease or even stop diabetes development. Nowadays, medicine emphasizes the need to understand the pathophysiological background of the disease in order to target treatment. There is a strong relationship between molecular pathophysiology and modifiable factors of glucose level—summary in Table 2 and Figure 1. The molecular knowledge of the disease allows for proposing small but particular interventions that are far more effective. Unfortunately, many of the studies cited are of value in the context of the future, with more research than specific therapeutic measures with the current state of the knowledge.

In type 2 diabetes, patients mainly struggle with the problem of hyperglycemia, however, an equally important and often overlooked aspect is the presence of hypoglycemia in them. As this work shows, so far, few studies have focused on the impact of too-low blood sugar levels on changes taking place in the cell, despite the significant clinical problems presented by patients with hypoglycemia, ranging from impaired consciousness and cognitive functions to even death. This indicates the need for further research on this aspect.

## Figures and Tables

**Table 2:** Factors affecting glucose fluctuations in the context of molecular discoveries.

Author	Years	Factors Affecting Glucose Level in the Context of Molecular Discoveries	Influence on Glucose Level ↑ Increase ↓ Decrease
Body weight			
Ying, W. et al. [12]	2019	➢ weight gain increased local proliferation of macrophages in islets that provide signals of promotion β-cell hyperplasia	↑
		➢ macrophages impair the ability to secrete insulin by β cells, enhancing insulin resistance and leading to hyperglycemia and exposure to lipotoxicity, causing frequent glucose variability	↑
		➢ by reducing inflammation, the effect of reducing glycemic fluctuations helps patients to reduce body weight	↓
Nagao, M. et al. [14]	2020	➢ weight gain influence on the overexpression of translocase CD36 in islets, which led to decreased insulin secretion, impaired exocytosis, and reduced granule docking	↑
		➢ CD36 antibody treatment of the human β-cell line EndoC-βH1 improves insulin secretion	↓
Zuo, D et al. [48]	2020	➢ obesity impacts the ability to secret insulin. Impaired secretion leads to insulin deficiency and, in consequence, glucose variability	↑
		➢ the newest obesity treatment intervention is bariatric surgery	↓
		➢ reduction in body mass and blood glucose levels, leading to a lower burden on β-cells and supporting the function of pancreatic islets bringing a positive impact on reducing the glucose level	↓
		➢ after three years follow-up, weight gain was observed on average by 31%	↑
Salehi, M et al. [15]	2018	➢ low blood sugar after RYGB surgery is explained by a wider glycemic excursion after food consumption, with an earlier and higher glucose peak and a lower glucose nadir as a result of bypassing the pylorus and the proximal intestine	↓
Dai, W et al. [21]	2021	➢ CaMKK adipocyte regulates insulin signaling and glucose homeostasis throughout the body ➢ CaMKK is involved in lipolysis and adipocyte inflammation, which may further contribute to the pathogenesis of insulin resistance ➢ adipocyte-specific inhibition of CAMKK may benefit metabolic dysfunctions such as hyperglycemia in type 2 diabetes	↓
Smoking			
Kim, J.H et al. [68]	2019	➢ the duration of exposure and the severity of nicotinism influence the processes of pancreatic β-cell dysfunction, causing glucose variability	↑
Sun, L et al. [22]	2020	➢ nicotine determinates aging of β cells of the pancreas both in vivo and in vitro; effect is impaired glucose metabolism	↑
		➢ the expression of senescent markers and accumulation of ROS in β cells revealed that nicotine intake enhances the senescence of β cells	↑
		➢ medical intervention that protects β cells from aging may be a strategy in the prevention of smoking-associated diabetes development, which is compatible with reducing glucose variability and maintaining metabolic homeostasis	↓
Duncan, A., et al. [23]	2019	➢ gene TCF7L2 inhibition is responsible for increasing nicotine consumption in rodents, while nicotine increases levels of blood glucose by TCF7L2-dependent stimulation of the mHb	↑
		➢ the pancreas has a polysynaptic connection to the mHb, so nicotine intake correlates with increased glucose and insulin level, which is similar to deregulating homeostasis due to T2DM development	↑
		➢ increased circulating glucose levels can modulate mHb function	↑
		➢ nicotine-induced increases in blood glucose may affect the response of mHb neurons and stop local nAChR function while causing habitual tobacco smoking	↑
		➢ deficiency in TCF7L2 dependent signaling leads to increased risk of tobacco addiction but simultaneously protects against smoking-related diabetes	↓
		➢ glucose variability affects the communication pathways between the pancreas and mHb, exacerbating the problem of smoking and decompensation of the underlying disease	↑
		➢ there are new perspectives for research to protect patients against nicotine-induced changes in habenula-regulated interactions with the anatomic nervous system	↓
Physical activity			
Ato, S et al. [82]	2019	➢ the number of muscle nuclei and mTORC1 activity deteriorated in the T2DM rats compared to the control group; the muscle mass affected by resistant training RT was similar to the healthy group	↓
		➢ physical exercise alters muscle mass and reduces the devastating effect of T2DM	↓
Krause, M et al. [24]	2015	➢ HSP70 has a significant role in the pathogenesis of insulin resistance	↑
		➢ reduced HSP70 concentration can inhibit inflammation and mitochondria fatty acid oxidation, and increase activation of SREBP-1c, which is a gene transcription factor in ER stress	↓
		➢ the elevation of the plasma levels of extracellular HSP70 is connected to obesity and diabetes, which are pro-inflammatory states	↑
		➢ increased HSP70 expression in brain cells can increase insulin sensitivity, normalize blood glucose level	↓
		➢ long-term exercise increases the production of intracellular HSP70 in the muscle cells of the liver, kidneys, and heart	↓
		➢ long-term exercises increase insulin sensitivity and decrease glucose variability, and make the constant metabolic conditions a beneficial effect on whole-body homeostasis	↓
Tsuzuki, T. et al. [27]	2017	➢ intracellular HSP72 is anti-inflammatory by blocking the activity of the JNK and NK-κB pathways and reducing the pro-inflammatory cytokines, increasing insulin sensitivity	↓
		➢ HSP72 has exercise-induced expression	↓
		➢ the concentration of HSP72 is decreased in skeletal muscle and liver in patients who suffer from obesity and insulin resistance	↑
		➢ well-chosen exercises maintain an appropriately high temperature of the patient’s body, ensuring an increase in HSP72. It is possible to generate appropriate metabolic pathways that will reduce the adverse effects of both obesity and T2DM, reducing glucose fluctuations	↓
Pearen, M.A et al. [90]	2018	➢ Nur77 and NOR1 induce insulin response genes and glucose and fat metabolism	↓
		➢ aerobic exercise, which offers the benefit of increasing insulin sensitivity, glucose, and lipid metabolism, may be related to regulation by NOR1	↓
Mey, J.T. et al. [30]	2019	➢ aerobic exercise strongly increases Nur77 and NOR1 in a healthy population	↓
		➢ obesity and in T2DM, the insulin response regulated by Nur77 and NOR1 is impaired, and interventions in the form of aerobic exercise partially restore this response	↓
		➢ there is a possibility of pharmacological enhancement of NOR1 while enhancing the positive effect of exercise on diabetes-related hyperglycemia	↓
Orumiyehei, A. et al. [32]	2022	➢ HIIT significantly reduced blood glucose and tau and Aβ protein levels	↓
Diet			
do Amaral, M.E.C. et al. [36]	2020	➢ lipotoxicity can disrupt Cx36 gap junction couplings within the islets in diet-induced obesity	↑
		➢ caloric restriction can protect against a decrease in insulin secretion and variability in glycemic homeostasis, for which intercellular communication inside the islets of Langerhans via the Cx36 gap junctions is responsible	↓
		➢ a decrease in Cx36 gap couplings that typically occurs in metabolic diseases can be regenerated by caloric restriction, improving Ca^2+^ dynamics and regulation of insulin secretion	↓
Liu, P. et al. [101]	2022	➢ HFD stimulates diabetes and insulin resistance in neuronal Thy1-C/EBPβ (Tg) transgenic mice, accompanied by a marked accumulation of mouse Aβ and hyperphosphorylated Tau aggregation in the brain	-
Arruda, A.P. et al. [37]	2011	➢ HFD causes attenuation of β-cell insulin secretion and peripheral insulin action through inflammation of the hypothalamus	↑
Tersey, S.A. et al. [103]	2014	➢ HFDs can activate 12-lipoxygenase activity in β-cells through cytokines, FFA, and increased blood glucose levels, thereby increasing the production of 12-hydroxyeicosatetraenoic acid (12-HETE)	↑
Johnston-Cox, H. et al. [38]	2012	➢ recent studies have shown that the A2b adenosine receptor (A2bAR), a recognized mediator of inflammation, controls pancreatic dysfunction in HFD-induced obesity	↑
Leri, M. et al. [34]	2019	➢ the polyphenols found in virgin olive oil have antioxidant properties and the ability to inhibit the growth of hIAPP, which is probably responsible for the loss of β-cell function and death	↓
von Hanstein, A.-S et al. [115]	2020	➢ the more significant component of unsaturated FFA in oil compositions does not have a protective factor against T2DM. Only the chain length of the FFA accounts for the toxicity of the FFA to EndoC-βH1 cells	↑
		➢ medium-chain FFA shorter than C16 is not toxic to EndoC-βH1 cells	-
		➢ the unique positive effect of consuming olive oil is not associated with a large amount of unsaturated fatty acids but with the presence of other plant components, such as polyphenols	↓
		➢ dietary recommendations in the form of consumption of olive oil are still valid and are justified by the protection of pancreatic cells against death, and thus worse glycemic homeostasis and frequent fluctuations leading to the diagnosis of T2DM	↓
Her, T.K. et al. [119]	2020	➢ significant reduction in carbohydrates in the diet has an impact on molecular changes in pancreatic islets, highlighted by the restricted capacity for beta cell mass expansion in response to HFD, which is characterized by a change in the secretory response	↓

## Data Availability

Data sharing not applicable. No new data were created or analyzed in this study. Data sharing is not applicable to this article.

## Abbreviations

DM—Diabetes Mellitus, T2DM—Type 2 Diabetes Mellitus, T1DM—Type 1 Diabetes Mellitus, Hb1Ac—Haemoglobin A1c, AGE—Advanced Glycation End products, CVD—Cardiovascular Disease, CAD—Coronary Artery Disease, SFA—Saturated Fatty Acids, BMI—Body Mass Index, FA—Fatty Acids, Cx36—Connexin36, LCFA—long-chain Free Fatty Acids, NODM—New-Onset Diabetes Mellitus, ROS—Reactive Oxygen Species, HFD—High-fat Diet, NMR—Nicotine Metabolism Rate, Mb—medial Habenula, NAChR—Nicotinic Acetylcholine receptors, mTORC1—mammalian Target Of Rapamycin Complex 1, RT—Resistance Training, HSP—Heat Shock Protein, HSP70—Heat Shock Protein 70, ER—Endoplasmic Reticulum, HSP72—Heat Shock Protein 72, Hiapp—human Islet Amyloid Polypeptide, Ole—Oleuropein, FFA—Free Farry acids, RYGB—Roux-en-Y Gastric Bypass, GLP-1—Glucagon-like peptide-1, NADPH—Nicotinamide adenine dinucleotide phosphate, PARP-1—Poly (ADP-ribose) polymerase 1, MPT—mitochondrial permeability transition, HERG—Human ether-à-go-go-related gene, CAMKK—calcium/calmodulin dependent-protein kinase II, INSR—insulin receptor, STM1—stromal interaction molecule 1, ITPR—inositol triphosphate receptor, HIIT—high-intensity interval training, AD—Alzheimer’s disease, Drp1—dynamin-related protein 1, BNIP3—Bcl-2/adenovirus E1B 19-kDa-interacting protein-3, BBB—Blood–Brain Barrier, PDGFR-β—platelet-derived growth factor receptor-β, AMPK—AMP-activated protein kinase, NF-kB—nuclear factor-κB, LDL—low-density lipoprotein, HDL—high-density lipoprotein, SOD—superoxide dismutase, GPx—glutathione peroxidase, MDA—malondialdehyde, TNF-α—factor-α, IL-1β interleukin-1β, IL-6 interleukin-6, COX-2—cyclooxygenase 2, PGE2—prostaglandin E2, NOX—NADPH oxidase, PKC—protein kinase C, Nrf2—Nuclear factor erythroid 2-related factor 2, MMP9—Matrix Metalloproteinase-9, 12-HETE—12-hydroxyeicosatetraenoic acid, A2bAR—A2b adenosine receptor, hIAPP—human islet amyloid polypeptide.

## References

[1] World Health Organisation Diabetes-Key Facts. https://www.who.int/news-room/fact-sheets/detail/diabetes.

[2] Saeedi P., Petersohn I., Salpea P., Malanda B., Karuranga S., Unwin N., Colagiuri S., Guariguata L., Motala A.A., Ogurtsova K. (2019). Global and regional diabetes prevalence estimates for 2019 and projections for 2030 and 2045: Results from the International Diabetes Federation Diabetes Atlas, 9th edition. Diabetes Res. Clin. Pract..

[3] Polskie Towarzystwo Diabetologiczne (2020). Guidelines on the Managment of Diabetic Patients. https://cukrzyca.info.pl/zalecenia_kliniczne/2020_guidelines_on_the_management_of_diabetic_patients.

[4] Association A.D. (2021). 2. Classification and diagnosis of diabetes: Standards of medical care in diabetes-2021. Diabetes Care.

[5] Guthrie R.A., Guthrie D.W. (2004). Pathophysiology of Diabetes Mellitus. Crit. Care Nurs. Q..

[6] Pasquel F.J., Lansang M.C., Dhatariya K., Umpierrez G.E. (2021). Management of Diabetes and Hyperglycaemia in the Hospital. Lancet Diabetes Endocrinol..

[7] Lauffenburger J.C., Lewey J., Jan S., Lee J., Ghazinouri R., Choudhry N.K. (2020). Association of Potentially Modifiable Diabetes Care Factors with Glycemic Control in Patients with Insulin-Treated Type 2 Diabetes. JAMA Netw. Open.

[8] Khattab M., Khader Y.S., Al-Khawaldeh A., Ajlouni K. (2010). Factors Associated with Poor Glycemic Control among Patients with Type 2 Diabetes. J. Diabetes Complicat..

[9] Cisińska G., Moczulski D. (2013). Analiza Czynników Ryzyka Cukrzycy Na Podstawie Ankiety FINDRISC Analysis of Risk Factors of Diabetes Based on FINDRISC Survey. Hygeia Public Health.

[10] Aghili R., Polonsky W.H., Valojerdi A.E., Malek M., Keshtkar A.A., Esteghamati A., Heyman M., Khamseh M.E. (2016). Type 2 Diabetes: Model of Factors Associated with Glycemic Control. Can. J. diabetes.

[11] Böni-Schnetzler M., Meier D.T. (2019). Islet inflammation in type 2 diabetes. Semin. Immunopathol..

[12] Ying W., Lee Y.S., Dong Y., Seidman J.S., Yang M., Isaac R., Seo J.B., Yang B.H., Wollam J., Riopel M. (2019). Expansion of Islet-Resident Macrophages Leads to Inflammation Affecting β Cell Proliferation and Function in Obesity. Cell Metab..

[13] Ye R., Gordillo R., Shao M., Onodera T., Chen Z., Chen S., Lin X., SoRelle J.A., Li X., Tang M. (2018). Intracellular lipid metabolism impairs β cell compensation during diet-induced obesity. J. Clin. Investig..

[14] Nagao M., Esguerra J.L.S., Asai A., Ofori J.K., Edlund A., Wendt A., Sugihara H., Wollheim C.B., Oikawa S., Eliasson L. (2020). Potential protection against type 2 diabetes in obesity through lower CD36 expression and improved exocytosis in β-cells. Diabetes.

[15] Salehi M., Vella A., McLaughlin T., Patti M.E. (2018). Hypoglycemia After Gastric Bypass Surgery: Current Concepts and Controversies. J. Clin. Endocrinol. Metab..

[16] Isaev N.K., Stel’Mashuk E.V., Zorov D.B. (2007). Cellular Mechanisms of Brain Hypoglycemia. Biochemistry.

[17] Languren G., Montiel T., Julio-Amilpas A., Massieu L. (2013). Neuronal Damage and Cognitive Impairment Associated with Hypoglycemia: An Integrated View. Neurochem. Int..

[18] Ozcan L., Wong C.C.L., Li G., Xu T., Pajvani U., Park S.K.R., Wronska A., Chen B.X., Marks A.R., Fukamizu A. (2012). Calcium Signaling through CaMKII Regulates Hepatic Glucose Production in Fasting and Obesity. Cell Metab..

[19] Wang Y., Yan S., Xiao B., Zuo S., Zhang Q., Chen G., Yu Y., Chen D., Liu Q., Liu Y. (2018). Prostaglandin F2α Facilitates Hepatic Glucose Production Through CaMKIIγ/P38/FOXO1 Signaling Pathway in Fasting and Obesity. Diabetes.

[20] Perry R.J., Zhang D., Guerra M.T., Brill A.L., Goedeke L., Nasiri A.R., Rabin-Court A., Wang Y., Peng L., Dufour S. (2020). Glucagon Stimulates Gluconeogenesis by INSP3R1-Mediated Hepatic Lipolysis. Nature.

[21] Dai W., Choubey M., Patel S., Singer H.A., Ozcan L. (2021). Adipocyte CAMK2 Deficiency Improves Obesity-Associated Glucose Intolerance. Mol. Metab..

[22] Sun L., Wang X., Gu T., Hu B., Luo J., Qin Y., Wan C. (2020). Nicotine triggers islet β cell senescence to facilitate the progression of type 2 diabetes. Toxicology.

[23] Duncan A., Heyer M.P., Ishikawa M., Caligiuri S.P.B., Liu X.-A., Chen Z., Di Bonaventura M.V.M., Elayouby K.S., Ables J.L., Howe W.M. (2019). Habenular TCF7L2 links nicotine addiction to diabetes. Nature.

[24] Krause M., Bock P.M., Takahashi H.K., De Bittencourt P.I.H., Newsholme P. (2015). The regulatory roles of NADPH oxidase, intra- and extra-cellular HSP70 in pancreatic islet function, dysfunction and diabetes. Clin. Sci..

[25] Esser N., Legrand-Poels S., Piette J., Scheen A.J., Paquot N. (2014). Inflammation as a link between obesity, metabolic syndrome and type 2 diabetes. Diabetes Res. Clin. Pract..

[26] Noble E.G., Ho R., Dzialoszynski T. (2006). Exercise is the primary factor associated with Hsp70 induction in muscle of treadmill running rats. Acta Physiol..

[27] Tsuzuki T., Kobayashi H., Yoshihara T., Kakigi R., Ichinoseki-Sekine N., Naito H. (2017). Attenuation of exercise-induced heat shock protein 72 expression blunts improvements in whole-body insulin resistance in rats with type 2 diabetes. Cell Stress Chaperones.

[28] Wu X., Wang J., Cui X., Maianu L., Rhees B., Rosinski J., So W.V., Willi S.M., Osier M.V., Hill H.S. (2007). The effect of insulin on expression of genes and biochemical pathways in human skeletal muscle. Endocrine.

[29] Chao L.C., Wroblewski K., Ilkayeva O.R., Stevens R.D., Bain J., Meyer G.A., Schenk S., Martinez L., Vergnes L., Narkar V.A. (2012). Skeletal muscle Nur77 expression enhances oxidative metabolism and substrate utilization. J. Lipid Res..

[30] Mey J.T., Solomon T.P.J., Kirwan J.P., Haus J.M. (2019). Skeletal muscle Nur77 and NOR1 insulin responsiveness is blunted in obesity and type 2 diabetes but improved after exercise training. Physiol. Rep..

[31] Castellani R.J., Rolston R.K., Smith M.A. (2010). Alzheimer Disease. Dis. Mon..

[32] Orumiyehei A., Khoramipour K., Rezaei M.H., Madadizadeh E., Meymandi M.S., Mohammadi F., Chamanara M., Bashiri H., Suzuki K. (2022). High-Intensity Interval Training-Induced Hippocampal Molecular Changes Associated with Improvement in Anxiety-like Behavior but Not Cognitive Function in Rats with Type 2 Diabetes. Brain Sci..

[33] Shai I., Schwarzfuchs D., Henkin Y., Shahar D.R., Witkow S., Greenberg I., Golan R., Fraser D., Bolotin A., Vardi H. (2008). Weight Loss with a Low-Carbohydrate, Mediterranean, or Low-Fat Diet. N. Engl. J. Med..

[34] Leri M., Natalello A., Bruzzone E., Stefani M., Bucciantini M. (2019). Oleuropeinaglycone and hydroxytyrosol interfere differently with toxic Aβ 1-42 aggregation. Food Chem. Toxicol..

[35] Stefani M., Rigacci S. (2013). Protein Folding and Aggregation into Amyloid: The Interference by Natural Phenolic Compounds. Int. J. Mol. Sci..

[36] do Amaral M.E.C., Kravets V., Dwulet J.A.M., Farnsworth N.L., Piscopio R., Schleicher W.E., Miranda J.G., Benninger R.K.P. (2020). Caloric restriction recovers impaired b-cell-b-cell gap junction coupling, calcium oscillation coordination, and insulin secretion in prediabetic mice. Am. J. Physiol.-Endocrinol. Metab..

[37] Arruda A.P., Milanski M., Coope A., Torsoni A.S., Ropelle E., Carvalho D.P., Carvalheira J.B., Velloso L.A. (2011). Low-Grade Hypothalamic Inflammation Leads to Defective Thermogenesis, Insulin Resistance, and Impaired Insulin Secretion. Endocrinology.

[38] Johnston-Cox H., Koupenova M., Yang D., Corkey B., Gokce N., Farb M.G., LeBrasseur N., Ravid K. (2012). The A2b Adenosine Receptor Modulates Glucose Homeostasis and Obesity. PLoS ONE.

[39] Krishan P., Bedi O., Rani M. (2018). Impact of diet restriction in the management of diabetes: Evidences from preclinical studies. Naunyn. Schmiedebergs. Arch. Pharmacol..

[40] Lee Y.S., Wollam J., Olefsky J.M. (2018). An Integrated View of Immunometabolism. Cell.

[41] Butcher M.J., Hallinger D., Garcia E., Machida Y., Chakrabarti S., Nadler J., Galkina E.V., Imai Y. (2014). Association of proinflammatory cytokines and islet resident leucocytes with islet dysfunction in type 2 diabetes. Diabetologia.

[42] Ye R., Wang M., Wang Q.A., Scherer P.E. (2015). Adiponectin-mediated antilipotoxic effects in regenerating pancreatic islets. Endocrinology.

[43] Ying W., Fu W., Lee Y.S., Olefsky J.M. (2020). The role of macrophages in obesity-associated islet inflammation and β-cell abnormalities. Nat. Rev. Endocrinol..

[44] Rosengren A.H., Braun M., Mahdi T., Andersson S.A., Travers M.E., Shigeto M., Zhang E., Almgren P., Ladenvall C., Axelsson A.S. (2012). Reduced insulin exocytosis in human pancreatic β-cells with gene variants linked to type 2 diabetes. Diabetes.

[45] Noushmehr H., D’Amico E., Farilla L., Hui H., Wawrowsky K.A., Mlynarski W., Doria A., Abumrad N.A., Perfetti R. (2005). Fatty acid translocase (FAT/CD36) is localized on insulin-containing granules in human pancreatic β-cells and mediates fatty acid effects on insulin secretion. Diabetes.

[46] Carlsson C., Borg L.A.H., Welsh N. (1999). Sodium palmitate induces partial mitochondrial uncoupling and reactive oxygen species in rat pancreatic islets in vitro. Endocrinology.

[47] Dixon J.B., Blazeby J.M. (2014). Quality of life after bariatric surgery. Lancet Diabetes Endocrinol..

[48] Zuo D., Xiao X., Yang S., Gao Y., Wang G., Ning G. (2020). Effects of bariatric surgery in Chinese with obesity and type 2 diabetes mellitus: A 3-year follow-up. Medicine.

[49] Oh T.J., Moon J.H., Choi S.H., Lim S., Park K.S., Cho N.H., Jang H.C. (2019). Body-Weight Fluctuation and Incident Diabetes Mellitus, Cardiovascular Disease, and Mortality: A 16-Year Prospective Cohort Study. J. Clin. Endocrinol. Metab..

[50] Yeboah P., Hsu F.C., Bertoni A.G., Yeboah J. (2019). Body Mass Index, Change in Weight, Body Weight Variability and Outcomes in Type 2 Diabetes Mellitus [from the ACCORD Trial]. Am. J. Cardiol..

[51] Salehi M., Gastaldelli A., D’Alessio D.A. (2014). Altered Islet Function and Insulin Clearance Cause Hyperinsulinemia in Gastric Bypass Patients with Symptoms of Postprandial Hypoglycemia. J. Clin. Endocrinol. Metab..

[52] Salehi M., Gastaldelli A., D’Alessio D.A. (2014). Blockade of Glucagon-like Peptide 1 Receptor Corrects Postprandial Hypoglycemia after Gastric Bypass. Gastroenterology.

[53] Salehi M., D’Alessio D.A. (2014). Effects of Glucagon like Peptide-1 to Mediate Glycemic Effects of Weight Loss Surgery. Rev. Endocr. Metab. Disord..

[54] Laferrère B., Teixeira J., McGinty J., Tran H., Egger J.R., Colarusso A., Kovack B., Bawa B., Koshy N., Lee H. (2008). Effect of Weight Loss by Gastric Bypass Surgery versus Hypocaloric Diet on Glucose and Incretin Levels in Patients with Type 2 Diabetes. J. Clin. Endocrinol. Metab..

[55] Sang W.S., Hamby A.M., Swanson R.A. (2007). Hypoglycemia, Brain Energetics, and Hypoglycemic Neuronal Death. Glia.

[56] Nordin C. (2014). The Proarrhythmic Effect of Hypoglycemia: Evidence for Increased Risk from Ischemia and Bradycardia. Acta Diabetol..

[57] Zhang Y., Han H., Wang J., Wang H., Yang B., Wang Z. (2003). Impairment of Human Ether-à-Go-Go-Related Gene (HERG) K+ Channel Function by Hypoglycemia and Hyperglycemia. Similar Phenotypes but Different Mechanisms. J. Biol. Chem..

[58] Priori S.G., Corr P.B. (1990). Mechanisms Underlying Early and Delayed Afterdepolarizations Induced by Catecholamines. Am. J. Physiol..

[59] Okazaki O., Suda N., Hongo K., Konishi M., Kurihara S. (1990). Modulation of Ca2+ Transients and Contractile Properties by Beta-Adrenoceptor Stimulation in Ferret Ventricular Muscles. J. Physiol..

[60] Choi B.R., Burton F., Salama G. (2002). Cytosolic Ca^2+^ Triggers Early Afterdepolarizations and Torsade de Pointes in Rabbit Hearts with Type 2 Long QT Syndrome. J. Physiol..

[61] Chen J., Fleming T., Katz S., Dewenter M., Hofmann K., Saadatmand A., Kronlage M., Werner M.P., Pokrandt B., Schreiter F. (2021). CaM Kinase II-δ Is Required for Diabetic Hyperglycemia and Retinopathy but Not Nephropathy. Diabetes.

[62] Segal S., Lloyd S., Sherman N., Sussman K., Draznin B. (1990). Postprandial Changes in Cytosolic Free Calcium and Glucose Uptake in Adipocytes in Obesity and Non-Insulin-Dependent Diabetes Mellitus. Horm. Res..

[63] Baumbach J., Hummel P., Bickmeyer I., Kowalczyk K.M., Frank M., Knorr K., Hildebrandt A., Riedel D., Jäckle H., Kühnlein R.P. (2014). A Drosophila in Vivo Screen Identifies Store-Operated Calcium Entry as a Key Regulator of Adiposity. Cell Metab..

[64] Subramanian M., Metya S.K., Sadaf S., Kumar S., Schwudke D., Hasan G. (2013). Altered Lipid Homeostasis in Drosophila InsP3 Receptor Mutants Leads to Obesity and Hyperphagia. DMM Dis. Model. Mech..

[65] Willi C., Bodenmann P., Ghali W.A., Faris P.D., Cornuz J. (2007). Active smoking and the risk of type 2 diabetes: A systematic review and meta-analysis. J. Am. Med. Assoc..

[66] Chang S.A. (2012). Smoking and type 2 diabetes mellitus. Diabetes Metab. J..

[67] Akter S., Goto A., Mizoue T. (2017). Smoking and the risk of type 2 diabetes in Japan: A systematic review and meta-analysis. J. Epidemiol..

[68] Kim J.H., Seo D.C., Kim B.J., Kang J.G., Lee S.J., Lee S.H., Kim B.S., Kang J.H. (2019). Association between cigarette smoking and new-onset diabetes mellitus in 78,212 Koreans using self-reported questionnaire and urine cotinine. Diabetes Metab. J..

[69] Epifano L., Di Vincenzo A., Fanelli C., Porcellati E., Perriello G., De Feo P., Motolese M., Brunetti P., Bolli G.B. (1992). Effect of cigarette smoking and of a transdermal nicotine delivery system on glucoregulation in type 2 diabetes mellitus. Eur. J. Clin. Pharmacol..

[70] Maddatu J., Anderson-Baucum E., Evans-Molina C. (2017). Smoking and the risk of type 2 diabetes. Transl. Res..

[71] Wang P., Fiaschi-Taesch N.M., Vasavada R.C., Scott D.K., García-Ocaña A., Stewart A.F. (2015). Diabetes mellitus-advances and challenges in human β-cell proliferation. Nat. Rev. Endocrinol..

[72] Pan A., Wang Y., Talaei M., Hu F.B., Wu T. (2015). Relation of active, passive, and quitting smoking with incident type 2 diabetes: A systematic review and meta-analysis. Lancet Diabetes Endocrinol..

[73] Goya Wannamethee S., Gerald Shaper A., Perry I.J. (2001). Smoking as a modifiable risk factor for type 2 diabetes in middle-aged men. Diabetes Care.

[74] Keith R.J., Riggs D.W., Conklin D.J., Lorkiewicz P., Srivastava S., Bhatnagar A., Defilippis A.P. (2018). Nicotine metabolism in adults with type 2 diabetes. Nicotine Tob. Res..

[75] Chiolero A., Faeh D., Paccaud F., Cornuz J. (2008). Consequences of smoking for body weight, body fat distribution, and insulin resistance. Am. J. Clin. Nutr..

[76] Adeghate E., Schattner P., Dunn E. (2006). An update on the etiology and epidemiology of diabetes mellitus. Ann. New York Acad. Sci..

[77] Srikanthan P., Karlamangla A.S. (2011). Relative muscle mass is inversely associated with insulin resistance and prediabetes. Findings from the Third National Health and Nutrition Examination Survey. J. Clin. Endocrinol. Metab..

[78] Damas F., Phillips S., Vechin F.C., Ugrinowitsch C. (2015). A Review of Resistance Training-Induced Changes in Skeletal Muscle Protein Synthesis and Their Contribution to Hypertrophy. Sport. Med..

[79] Peterson J.M., Bryner R.W., Alway S.E. (2008). Satellite cell proliferation is reduced in muscles of obese Zucker rats but restored with loading. Am. J. Physiol.-Cell Physiol..

[80] Katta A., Kundla S., Kakarla S.K., Wu M., Fannin J., Paturi S., Liu H., Addagarla H.S., Blough E.R. (2010). Impaired overload-induced hypertrophy is associated with diminished mTOR signaling in insulin-resistant skeletal muscle of the obese Zucker rat. Am. J. Physiol.-Regul. Integr. Comp. Physiol..

[81] Williamson D.L., Li Z., Tuder R.M., Feinstein E., Kimball S.R., Dungan C.M. (2014). Altered nutrient response of mTORC1 as a result of changes in REDD1 expression: Effect of obesity vs. REDD1 deficiency. J. Appl. Physiol..

[82] Ato S., Kido K., Sato K., Fujita S. (2019). Type 2 diabetes causes skeletal muscle atrophy but does not impair resistance training-mediated myonuclear accretion and muscle mass gain in rats. Exp. Physiol..

[83] Lindström J., Ilanne-Parikka P., Peltonen M., Aunola S., Eriksson J.G., Hemiö K., Hämäläinen H., Härkönen P., Keinänen-Kiukaanniemi S., Laakso M. (2006). Sustained reduction in the incidence of type 2 diabetes by lifestyle intervention: Follow-up of the Finnish Diabetes Prevention Study. Lancet.

[84] Lee D.-c., Park I., Jun T.-W., Nam B.-H., Cho S.-i., Blair S.N., Kim Y.-S. (2012). Physical Activity and Body Mass Index and Their Associations with the Development of Type 2 Diabetes in Korean Men. Am. J. Epidemiol..

[85] Geiger P.C., Gupte A.A. (2011). Heat shock proteins are important mediators of skeletal muscle insulin sensitivity. Exerc. Sport Sci. Rev..

[86] Tytell M., Davis A.T., Giles J., Snider L.C., Xiao R., Dozier S.G., Presley T.D., Kavanagh K. (2018). Alfalfa-derived HSP70 administered intranasally improves insulin sensitivity in mice. Cell Stress Chaperones.

[87] Mulyani W.R.W., Sanjiwani M.I.D., Sandra, Prabawa P.Y., Lestari A.A.W., Wihandani D.M., Suastika K., Saraswati M.R., Bhargah A., Manuaba I.B.A.P. (2020). Chaperone-based therapeutic target innovation: Heat shock protein 70 [HSP70] for type 2 diabetes mellitus. Diabetes Metab. Syndr. Obes. Targets Ther..

[88] Chung J., Nguyen A.K., Henstridge D.C., Holmes A.G., Chan M.H.S., Mesa J.L., Lancaster G.I., Southgate R.J., Bruce C.R., Duffy S.J. (2008). HSP72 protects against obesity-induced insulin resistance. Proc. Natl. Acad. Sci. USA.

[89] Atkin A.S., Moin A.S.M., Nandakumar M., Al-Qaissi A., Sathyapalan T., Atkin S.L., Butler A.E. (2021). Impact of Severe Hypoglycemia on the Heat Shock and Related Protein Response. Sci. Rep..

[90] Pearen M.A., Muscat G.E.O. (2018). The Nuclear Receptor Nor-1 Is a Pleiotropic Regulator of Exercise-Induced Adaptations. Exerc. Sport Sci. Rev..

[91] Nisar O., Pervez H., Mandalia B., Waqas M., Sra H.K. (2020). Type 3 Diabetes Mellitus: A Link Between Alzheimer’s Disease and Type 2 Diabetes Mellitus. Cureus.

[92] Anderson R.J., Freedland K.E., Clouse R.E., Lustman P.J. (2001). The Prevalence of Comorbid Depression in Adults with Diabetes: A Meta-Analysis. Diabetes Care.

[93] Mikaliukštiene A., Žagminas K., Juozulynas A., Narkauskaite L., Salyga J., Jankauskiene K., Stukas R., Šurkiene G. (2014). Prevalence and Determinants of Anxiety and Depression Symptoms in Patients with Type 2 Diabetes in Lithuania. Med. Sci. Monit..

[94] Raimundo A.F., Ferreira S., Martins I.C., Menezes R. (2020). Islet Amyloid Polypeptide: A Partner in Crime with Aβ in the Pathology of Alzheimer’s Disease. Front. Mol. Neurosci..

[95] Zhou Y., Huang L., Zheng W., An J., Zhan Z., Wang L., Chen Z., Liu L. (2018). Recurrent Nonsevere Hypoglycemia Exacerbates Imbalance of Mitochondrial Homeostasis Leading to Synapse Injury and Cognitive Deficit in Diabetes. Am. J. Physiol. Endocrinol. Metab..

[96] Lin L., Wu Y., Chen Z., Huang L., Wang L., Liu L. (2021). Severe Hypoglycemia Contributing to Cognitive Dysfunction in Diabetic Mice Is Associated with Pericyte and Blood–Brain Barrier Dysfunction. Front. Aging Neurosci..

[97] Esposito K., Chiodini P., Maiorino M.I., Bellastella G., Panagiotakos D., Giugliano D. (2014). Which diet for prevention of type 2 diabetes? A meta-analysis of prospective studies. Endocrine.

[98] Forouhi N.G., Misra A., Mohan V., Taylor R., Yancy W. (2018). Dietary and nutritional approaches for prevention and management of type 2 diabetes. BMJ.

[99] Imai Y., Cousins R.S., Liu S., Phelps B.M., Promes J.A. (2019). Connecting pancreatic islet lipid metabolism with insulin secretion and the development of type 2 diabetes. Ann. N. Y. Acad. Sci..

[100] Duan Y., Zeng L., Zheng C., Song B., Li F., Kong X., Xu K. (2018). Inflammatory Links Between High Fat Diets and Diseases. Front. Immunol..

[101] Liu P., Wang Z.H., Kang S.S., Liu X., Xia Y., Chan C.B., Ye K. (2022). High-Fat Diet-Induced Diabetes Couples to Alzheimer’s Disease through Inflammation-Activated C/EBPβ/AEP Pathway. Mol. Psychiatry.

[102] Li H., Meng Y., He S., Tan X., Zhang Y., Zhang X., Wang L., Zheng W. (2022). Macrophages, Chronic Inflammation, and Insulin Resistance. Cells.

[103] Tersey S.A., Maier B., Nishiki Y., Maganti A.V., Nadler J.L., Mirmira R.G. (2014). 12-Lipoxygenase Promotes Obesity-Induced Oxidative Stress in Pancreatic Islets. Mol. Cell. Biol..

[104] Rui L. (2014). Energy Metabolism in the Liver. Compr. Physiol..

[105] Ghazala R.A., El Medney A., Meleis A., Mohie El dien P., Samir H. (2020). Role of anti-inflammatory interventions in high-fat-diet-induced obesity. Biomed. Chromatogr..

[106] Elhayany A., Lustman A., Abel R., Attal-Singer J., Vinker S. (2010). A low carbohydrate Mediterranean diet improves cardiovascular risk factors and diabetes control among overweight patients with type 2 diabetes mellitus: A 1-year prospective randomized intervention study. Diabetes Obes. Metab..

[107] Esposito K., Giugliano D. (2014). Mediterranean diet and type 2 diabetes. Diabetes Metab. Res. Rev..

[108] Nani A., Murtaza B., Khan A.S., Khan N.A., Hichami A. (2021). Antioxidant and Anti-Inflammatory Potential of Polyphenols Contained in Mediterranean Diet in Obesity: Molecular Mechanisms. Molecules.

[109] Galic S., Fullerton M.D., Schertzer J.D., Sikkema S., Marcinko K., Walkley C.R., Izon D., Honeyman J., Chen Z.-P., Van Denderen B.J. (2011). Hematopoietic AMPK β1 reduces mouse adipose tissue macrophage inflammation and insulin resistance in obesity. J. Clin. Investig..

[110] Gauthier M.-S., O’Brien E.L., Bigornia S., Mott M., Cacicedo J.M., Xu X.J., Gokce N., Apovian C., Ruderman N. (2011). Decreased AMP-activated protein kinase activity is associated with increased inflammation in visceral adipose tissue and with whole-body insulin resistance in morbidly obese humans. Biochem. Biophys. Res. Commun..

[111] Ye L., Hu P., Feng L.-P., Huang L.-L., Wang Y., Yan X., Xiong J., Xia H.-L. (2022). Protective Effects of Ferulic Acid on Metabolic Syndrome: A Comprehensive Review. Molecules.

[112] Huang Y., Zhu X., Chen K., Lang H., Zhang Y., Hou P., Ran L., Zhou M., Zheng J., Yi L. (2019). Resveratrol prevents sarcopenic obesity by reversing mitochondrial dysfunction and oxidative stress via the PKA/LKB1/AMPK pathway. Aging.

[113] Obrenovich M., Li Y., Tayahi M., Reddy V.P. (2022). Polyphenols and Small Phenolic Acids as Cellular Metabolic Regulators. Curr. Issues Mol. Biol..

[114] Chaari A. (2020). Inhibition of human islet amyloid polypeptide aggregation and cellular toxicity by oleuropein and derivatives from olive oil. Int. J. Biol. Macromol..

[115] von Hanstein A.-S., Lenzen S., Plötz T. (2020). Toxicity of fatty acid profiles of popular edible oils in human EndoC-βH1 beta-cells. Nutr. Diabetes.

[116] Schwingshackl L., Lampousi A.M., Portillo M.P., Romaguera D., Hoffmann G., Boeing H. (2017). Olive oil in the prevention and management of type 2 diabetes mellitus: A systematic review and meta-analysis of cohort studies and intervention trials. Nutr. Diabetes.

[117] Mosser R.E., Maulis M.F., Moullé V.S., Dunn J.C., Carboneau B.A., Arasi K., Pappan K., Poitout V., Gannon M. (2015). High-fat diet-induced β-cell proliferation occurs prior to insulin resistance in C57Bl/6J male mice. Am. J. Physiol.-Endocrinol. Metab..

[118] Stamateris R.E., Sharma R.B., Kong Y., Ebrahimpour P., Panday D., Ranganath P., Zou B., Levitt H., Parambil N.A., O’Donnell C.P. (2016). Glucose Induces mouse β-cell proliferation via IRS2, MTOR, and cyclin D2 but Not the insulin receptor. Diabetes.

[119] Her T.K., Lagakos W.S., Brown M.R., LeBrasseur N.K., Rakshit K., Matveyenko A.V. (2020). Dietary carbohydrates modulate metabolic and β-cell adaptation to high-fat diet-induced obesity. Am. J. Physiol.-Endocrinol. Metab..

